# A phase Ib/II trial of atezolizumab with cobimetinib or idasanutlin in metastatic estrogen receptor positive breast cancer

**DOI:** 10.1038/s41523-025-00773-4

**Published:** 2025-06-21

**Authors:** Jessica Mezzanotte-Sharpe, Brandie C. Taylor, Paula I. Gonzalez-Ericsson, Violeta Sanchez, Andres A. Ocampo, Jacey L. Marshall, Julia A. Steele, Melinda E. Sanders, Ingrid A. Mayer, Justin M. Balko, Laura C. Kennedy

**Affiliations:** 1 Departments of Medicine, Vanderbilt University Medical Center, Nashville, TN USA; 2 Cancer Biology Program, Vanderbilt University, Nashville, TN USA; 3 Pathology, Microbiology, & Immunology, Vanderbilt University Medical Center, Nashville, TN USA

**Keywords:** Breast cancer, Cancer therapy, Cancer immunotherapy, Targeted therapies, Predictive markers

## Abstract

Despite the availability of numerous treatment options for metastatic estrogen receptor positive breast cancer, additional strategies are needed, particularly when tumors become endocrine resistant. This phase Ib/II study examined the clinical activity and safety of the novel combination of atezolizumab with molecularly targeted therapy inhibiting 1) the Ras/Raf/MEK signaling pathway with cobimetinib in *TP53*-mutant tumors (arm COBI) or 2) the TP53 regulator MDM2 with idasanutlin in *TP53-*wild-type tumors (arm IDA). Twelve patients were enrolled before the study closed early due to slow accrual. 2/7 patients in arm IDA had durable responses to treatment. 1/5 patients in arm COBI had stable disease. Interestingly, conservation of tumor-specific HLA-ABC expression was observed in nearly all patients with clinical benefit. There were several grade 3-4 toxicities, particularly cytopenias in arm IDA. While this study was limited by small sample sizes, there were observations of clinical activity, including one exceptional responder, that warrant further investigation.

## Introduction

Despite advances in early detection and therapeutic options, metastatic breast cancer (MBC) remains an incurable disease and is a leading cause of cancer-related mortality^1^. Breast cancer has three primary subtypes, the most common of which is positivity for expression of the estrogen (ER) and/or progesterone (PR) receptors. The mainstay of systemic therapy for patients with metastatic ER/PR+ breast cancer involves therapies that either antagonize estrogen binding to the ER, suppress and downregulate the ER or block estrogen biosynthesis. This is typically combined with targeted agents like cyclin-dependent kinase 4/6 (CDK4/6) inhibitors, PI3K inhibitors, or chemotherapy^2^. However, patients with ER + MBC inevitably develop acquired resistance to anti-estrogen therapy. Therefore, new strategies are needed to prolong survival in patients with ER + MBC^3^.

Immune checkpoint inhibition (ICI), a treatment approach using monoclonal antibodies to block immune checkpoint ligand/receptor interactions, has shown notable success in various cancer types and can induce durable responses^4^. While pembrolizumab (anti-PD-1) is now a standard of care in metastatic triple negative breast cancer (TNBC) patients with high combined positive scores (CPS)^5,6^, the data on ICIs in ER+ breast cancers are less promising, where most studies involve heavily pretreated patient populations with limited success^7–9^.

*TP53* is one of the most commonly mutated genes in breast cancer, and its status may provide insight into the mechanics of immune evasion. Both luminal B and basal-like breast cancer subtypes are enriched for *TP53* mutations^10–12^. Previous studies have noted that *TP53*-mutated tumors can have loss of Ras/MAPK negative regulators, which results in MEK activation and loss of antigen presentation^13,14^. The addition of MEK inhibition to anti-PD-1 therapy has demonstrated enhanced immune responses in breast tumors^15,16^. Conversely, *TP53* wild-type tumors have a lack of T cell recruitment. Rising levels of p53 would result in an induction of senescence, which is associated with recruitment of activated T cells and production of T-cell recruiting chemokines CCL5, CXCL9, and CXCL11^17–19^. However, wild-type p53 is negatively regulated by MDM2, which drives ubiquitination and proteasomal degradation of p53^20,21^, inhibiting the induction of the Senescence-Associated Secretory Program (SASP). Addition of an MDM2 antagonist prevents ubiquitination of p53, which in turn activates senescence and leads to an enhancement in the tumor microenvironment through T cell recruitment and chemokine production^20–22^. Based on these findings, we hypothesized that the effectiveness of ICIs in ER + MBC could be augmented either by 1) increasing antigen presentation through MEK inhibition in *TP53*-mutant tumors or 2) by enhancing T cell recruitment and chemokine expression through MDM2 inhibition in *TP53*-wild type tumors.

In this open-label, two-arm phase Ib/II study, we evaluated the anti-tumor effect of the anti-PD-L1 monoclonal antibody atezolizumab in combination with either the MEK inhibitor cobimetinib in patients with *TP53*-mutated ER + MBC (arm COBI) or with the MDM2 antagonist idasanutlin in patients with *TP53*-wild-type ER + MBC (arm IDA). The phase Ib portion aimed to determine safety and tolerability of atezolizumab and idasanutlin ER + MBC patients; there were pre-existing safety data for the combination of atezolizumab and cobimetinib^15^. The goal of the phase II portion of the study was to evaluate the anti-tumor response of atezoliumab with idasanutlin or cobimetinib. Due to low accrual and the COVID-19 pandemic, the study was closed early after enrolling 12 patients (target enrollment = 34 patients). Correlative studies examining antigen presentation, CD8 + T-cell recruitment, and T-cell homing cytokines in the tumor microenvironment were performed. Despite the limitations posed by the small sample size, the combination therapy did show clinical activity and trends in immune response signatures in several patients. These findings suggest that further exploration of MEK and/or MDM2 inhibition may be strategies to improve ICI response rates in ER + MBC patients.

## Results and discussion

### Patient demographics

Twelve eligible patients aged 39-72 (median age 60) were enrolled in the study. Patient demographics are displayed in Table 1. All patients enrolled were female, most were white non-Hispanic individuals, and all had visceral metastases at the time of study enrollment and ECOG performance status of 1. Five patients were enrolled in arm COBI, and 7 patients were enrolled in arm IDA based on *TP53* mutation status. Of note, one patient enrolled in the study (arm IDA) was originally noted as having a *TP53* mutation, but this was amended prior to treatment initiation after both a confirmatory next-generation sequencing report amendment and Guardant360 liquid biopsy did not show a *TP53* mutation. For correlative studies, biopsies were obtained from the following metastatic sites: bone (3 of 12 patients), lymph nodes (3 of 12 patients), and liver (6 of 12 patients).

**Table 1:** Patient Characteristics

Idasanutlin Arm	Total (*N* = 7)
**Age**	
Mean (SD)	56.7 (12.2)
Median [Min, Max]	58.0 [39.0, 72.0]
**Race**	
Unknown	2 (28.6%)
White	5 (71.4%)
**Idasanutlin Dose**	
Ida 100 mg	3 (42.9%)
Ida 150 mg	3 (42.9%)
Ida 50 mg	1 (14.3%)
**TP53 Mutation Status**	
No	7 (100.0%)
Yes	0 (0.0%)
**ER Percentage**	
0-10	0 (0.0%)
11-30	0 (0.0%)
31-100	7 (100.0%)
**PR Percentage**	
0-10	3 (42.9%)
11-30	3 (42.9%)
31-100	1 (14.3%)
**Prior Lines ET**	
Median (Range)	2 (2, 3)
**Prior Lines Chemotherapy**	
Median (Range)	1(0–3)

Cobimetinib Arm	Total (*N* = 5)
**Age**	
Mean (SD)	60.2 (9.20)
Median [Min, Max]	63.0 [46.0, 70.0]
**Race**	
Unknown	1 (20.0%)
White	4 (80.0%)
**Cobimetinib Dose**	
Cobi 60- > 40 mg	1 (20.0%)
Cobi 60 mg	4 (80.0%)
**TP53 Mutation Status**	
Yes	5 (100%)
No	0 (0.0%)
**ER Percentage**	
0–10	0 (0.0%)
11–30	1 (20.0%)
31–100	4 (80.0%)
**PR Percentage**	
0–10	3 (60.0%)
11–30	1 (20.0%)
31–100	1 (20.0%)
**Prior Lines ET**	
Median (Range)	1 (1, 2)
**Prior Lines Chemotherapy**	
Median (Range)	1 (1, 2)

Prior lines of therapy are also indicated with median number of lines of treatment as well as the range of number of lines of treatment. ER: estrogen receptor. PR: progesterone receptor. ET: endocrine-based therapy.

### Safety and tolerability of Atezolizumab + Idasanutlin

Adverse events were monitored and reported throughout the study. The total number of reported grade 3-4 adverse events for each treatment arm are shown in Table 2. There were no grade 5 toxicities reported. One patient in arm IDA stopped treatment due to a dose-limiting toxicity (DLT), grade 4 pancytopenia. One patient initially planned for dose level 1 of idasanutlin (100 mg) was started at dose level -1 (50 mg) due to concerns for potential toxicities due to pre-existing nausea. No other DLTs were reported, although grade 3-4 decreases in white blood cell count (WBC) were reported in both arms, with 4 events in arm IDA and one event in arm COBI. Due to low accrual, the MTD of idasanutlin, and therefore the recommended phase II dose, was unable to be assessed.

**Table 2:** Total number of reported grade 3-4 adverse events for patients in each treatment arm

Idasanutlin Arm Adverse Events	Total (*N* = 18)
**Event**	
Anemia	2 (11.1%)
Diarrhea	1 (5.6%)
Glucose intolerance	1 (5.6%)
Hypertension	1 (5.6%)
Infection	1 (5.6%)
Leukocytosis	1 (5.6%)
Nausea	1 (5.6%)
Nephrolithiasis	1 (5.6%)
Neutropenic fever	1 (5.6%)
Osteonecrosis of the jaw	1 (5.6%)
Skin and subcutaneous tissue disorders	1 (5.6%)
Thrombocytopenia	2 (11.1%)
WBC decreased	4 (22.2%)

Cobimetinib Arm Adverse Events	Total (*N* = 11)
**Event**	
Anorexia	1 (9.1%)
Arthralgia	1 (9.1%)
AST increase	1 (9.1%)
Back pain	1 (9.1%)
Diarrhea	1 (9.1%)
Fatigue	1 (9.1%)
Hypercalcemia	1 (9.1%)
Hypoalbuminemia	1 (9.1%)
Hypokalemia	1 (9.1%)
Infection	1 (9.1%)
WBC decreased	1 (9.1%)

### Safety of Atezolizumab + Cobimetinib

Grade 3-4 adverse events are reported in Table 2. There were no grade 5 toxicities reported. Overall, few adverse events were observed in this treatment arm, likely due to the small sample size (5 patients).

### Clinical activity

**Fig. 1:**
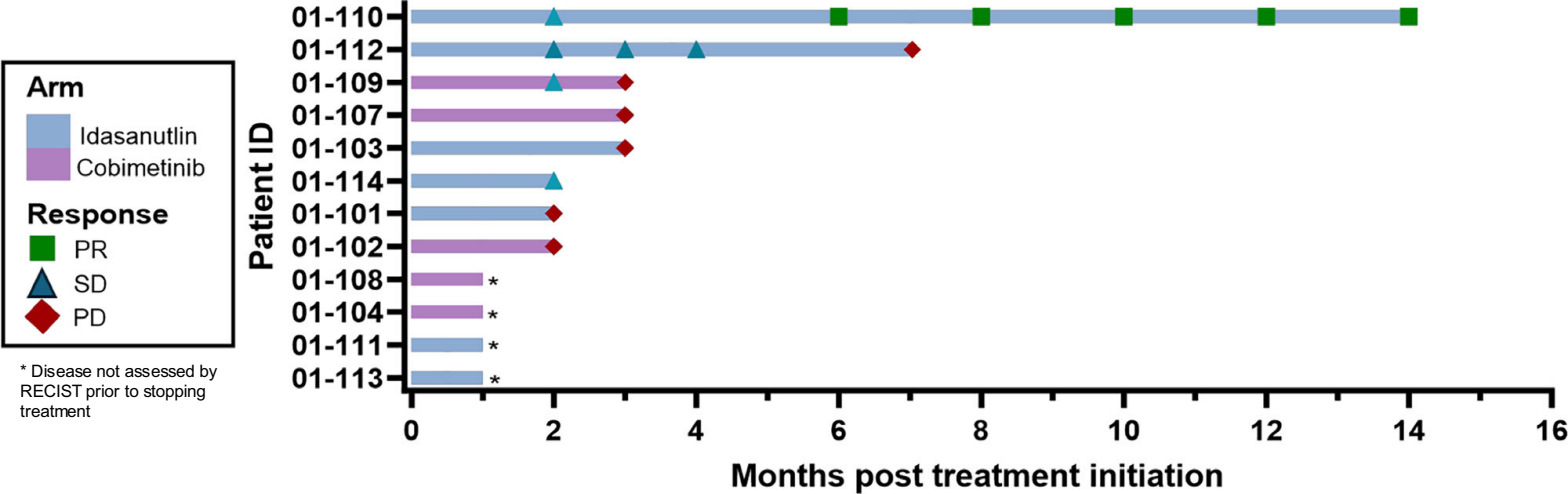
Progression-free survival. Swimmer plot showing months on treatment as well as response to treatment as determined by RECIST v1.1. Response to treatment is indicated at each time point it was taken. PR: Partial response, SD: Stable disease, PD: Progressive disease. * Disease not assessed by RECIST prior to stopping treatment.

PFS was a primary endpoint of this study. PFS at 16 weeks was the planned endpoint to determine if the study would move from stage I to stage II using Simon’s minmax design, but since this study closed early and did not accrue enough subjects to assess a stage I response, we report median PFS data here. PFS is shown for each study subject via swimmer plot in addition to disease data in Fig. 1. Median PFS was 2 months in both arms, ranging from 2 to 4 months in arm COBI and 1 to 14 months in arm IDA (two patients in arm IDA were removed from the study after one month of therapy due to concern for clinical progression of disease prior to planned study assessment). Two patients in arm IDA had disease control greater than 6 months. Both of those patients received idasanutlin at a 100 mg dose on days 1-5 of each cycle of treatment. Of note, the patient with an exceptional response of 14 months while enrolled in the study did not stop treatment due to progression of disease but instead due to a potential toxicity event. This patient continued to show stable disease for three years after discontinuation of trial treatment before progression.

Patient responses were measured using RECIST v1.1 for the secondary endpoint of objective response rate. For arm IDA, 1 out of 7 patients (14%) had a partial response (PR) to treatment, 2 had stable disease (SD), and 2 had progressive disease (PD). While the MTD of idasanutlin + atezolizumab was never established, it is important to note that none of the patients who received the highest dose of idasanutlin in this study, 150 mg, experienced a long-term response to treatment, and one in that group had a DLT (pancytopenia). In arm COBI, no patients had a PR or CR, 1 had SD, and 2 had PD. Four patients (2 in each arm) stopped treatment due to either toxicity or clinical progression of disease prior to having their response to treatment assessed by RECIST. Clinical benefit rate, defined as lack of disease progression at 6 months, was also limited by the small sample size and was 0 out of 5 patients in arm COBI and 2 out of 7 patients (28.5%) on arm IDA.

### Tumor biomarkers and molecular profiling

**Fig. 2:**
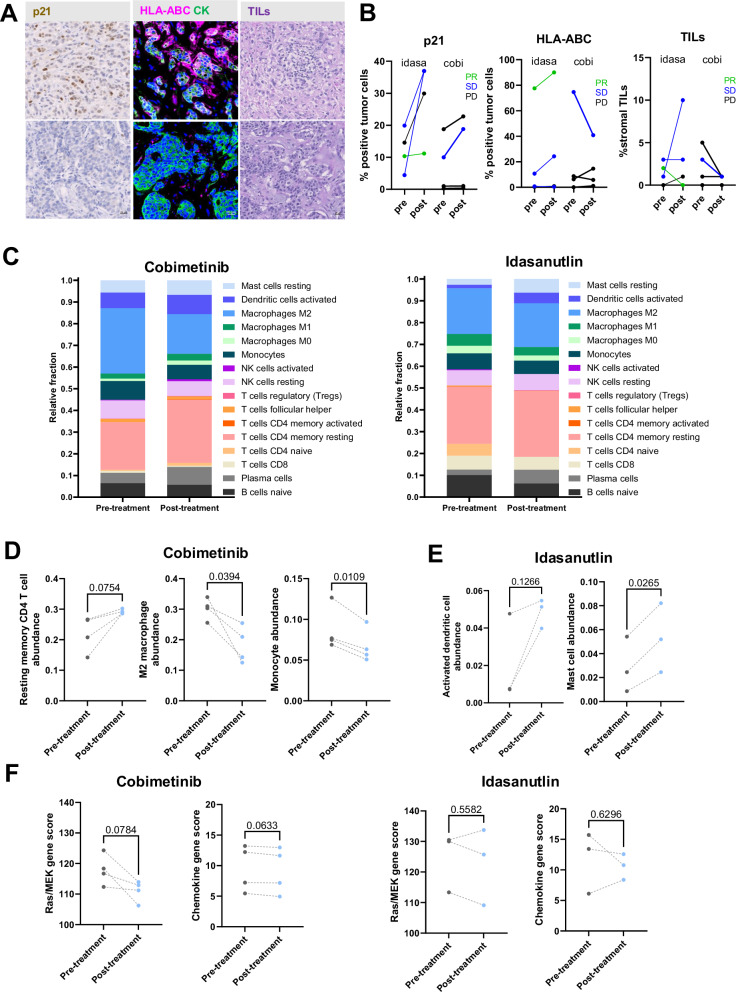
IHC and immune signature analysis. **A** p21 and HLA-ABC were evaluated via multiplexed IHC, and TILs were scored on H&E. Representative images for each assay. **B** Results are presented for each treatment arm pre- and post-treatment with idasanutlin (idasa) or cobimetinib (cobi). **C** Relative fractions of immune cells pre- and post-treatment with cobimetinib and idasanutlin. **D** Changes in resting memory CD4 T cell abundance, M2 macrophage abundance, and monocyte abundance with cobimetinib treatment. **E** Changes in activated dendritic cell abundance and mast cell abundance in the idasanutlin treatment arm. **F** Ras/MEK and chemokine gene score changes in patients before and after treatment with either cobimetinib or idasanutlin.

Tumor expression of p21 and HLA-A/B/C were evaluated via multiplexed IHC and TILs were scored on an H&E on samples procured prior and after treatment lead-in with each study agent. (Fig. 2A). p21 expression, a pharmacodynamic marker of p53 activity, increased after treatment with idasanutlin as expected, although these changes were not statistically significant (Fig. 2B*p* = 0.08). p21 expression was not changed in arm COBI. Tumor-specific HLA-A/B/C expression was substantially higher at baseline and slightly increased following treatment in arm IDA for the patient with PR. Stromal TILs were without significant changes following treatment with either idasanutlin or cobimetinib. There were very low levels of TILs at baseline in the examined samples, which is expected in patients with ER + MBC^23,24^.

RNA sequencing was used to examine gene expression patterns for MEK activation signatures as well as immune cell composition by CIBERSORT. This analysis was highly exploratory in terms of immune cell prediction and was limited by small sample size for each treatment group. Relative immune cell fractions and heat maps for each treatment arm are shown in Fig. 2C with notable trends shown in Fig. 2D, E. In the cobimetinib arm, there was a trend towards increases in resting memory CD4 T-cells (p = 0.0754), statistically significant decreases in M2 macrophage abundance (*p* = 0.0394), and statistically significant decreases in monocytes abundance (*p* = 0.0109) following treatment (Fig. 2D). In arm IDA, there was a trend towards increased activated dendritic cells in tumors and a statistically significant increase in mast cell abundance (*p* = 0.0265) following treatment (Fig. 2E). There was also an expected decrease, although not statistically significant, in Ras/MEK gene scores following cobimetinib treatment (*p* = 0.0784) that was not seen in arm IDA (Fig. 2F). Chemokine gene scores did not significantly change in either treatment arm (of note, chemokine gene scores were not validated for idasanutlin).

### Analysis of patient ctDNA

**Fig. 3:**
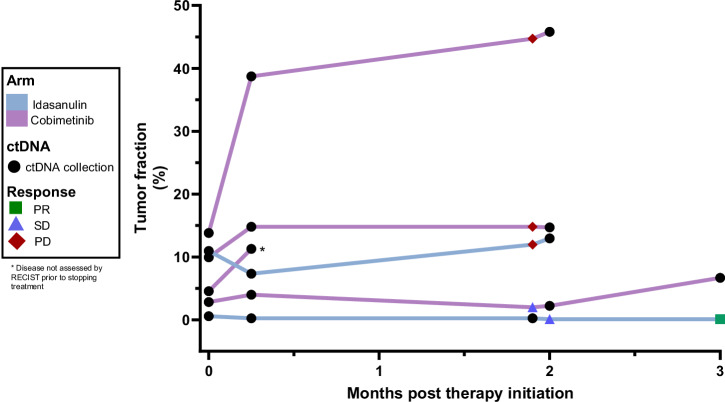
ctDNA analysis. Longitudinal follow up of ctDNA in patients in arm IDA and arm COBI. ctDNA is represented by percent of tumor fraction. Response to treatment is indicated at each time point it was taken. PR Partial response, SD Stable disease, PD Progressive disease.

In this study, we monitored circulating tumor DNA (ctDNA) levels in a subset of patients from arm IDA and arm COBI prior to treatment initiation and post-treatment. Six patients (2 from arm IDA and 4 from arm COBI) were analyzed. All patients had some level of detectable ctDNA at C1D1. Detectable ctDNA within one month after the initiation of therapy was strongly correlated with progressive disease, irrespective of the treatment arm (Fig. 3). The patient with the exceptional response, who maintained stable disease for three years before progression, consistently had the lowest levels of ctDNA among all patients. In contrast, patients experiencing progressive disease exhibited rising ctDNA levels over time, which corresponded closely with clinical indicators of disease progression.

## Discussion

Although ER + MBC patients have the best overall prognosis in terms of life expectancy among the different MBC subtypes^25^, opportunities for therapeutic advances remain. ICI therapy has not yet been approved in this group of patients outside the setting of high tumor mutational burden or microsatellite instability, but there are likely additional groups of ER+ mBC patients who could benefit with the correct combination of agents. This study aimed to examine the novel combination of ICIs with MEK inhibition in *TP53*-mutant tumors or with MDM2 antagonists in *TP53-*wt tumors in ER + MBC patients. Specifically, the phase Ib portion of this study assessed the safety and tolerability of combining atezolizumab+idasanutlin in *TP53*-wt patients, while the phase II portion aimed to measure the progression-free survival of combination atezolizumab+idasanutlin in *TP53*-wt patients and atezolizumab+cobimetinib in *TP53*-mutant patients.

Although this study was ultimately stopped early due to low patient accrual and the COVID-19 pandemic, limiting our ability to statistically analyze the PFS as originally planned, two patients in arm IDA remained on treatment without disease progression for over six months, and one patient in arm COBI had stable disease prior to progression. Notably, both patients with long-term responses in the atezolizumab+idasanutlin group received idasanutlin at a dose of 100 mg. It is also important to note that all patients enrolled in this study were heavily pre-treated.

While the phase Ib dose escalation portion of this study planned to have patients receive up to 200 mg of idasanutlin on days 1-5 of each treatment cycle, insufficient enrollment prevented completion of the phase Ib portion of the study. While one patient enrolled at the 150 mg dose experienced a DLT, additional patients were not enrolled at the 100 mg or 150 mg idasanutlin dose level to determine the maximum tolerated doses (MTD). This limitation necessitates further investigation to confirm the MTD for this agent in combination with atezolizumab, especially given the two patients with longer durations of treatment response at the 100 mg dose level. Regarding safety and tolerability, several grade 3-4 adverse events were observed in arm IDA, with cytopenia being the most common observed toxicity. No grade 5 adverse events were observed.

Cobimetinib in combination with atezolizumab was well-tolerated overall with reported adverse events consistent with the previously reported safety profile for these agents^26^. Only one patient on the study requiring a dose reduction from 60 mg to 40 mg, with diarrhea, nausea, and vomiting leading to grade 2 dehydration prior to the dose reduction. Future studies with larger sample sizes will be needed in the future for both agents to further examine toxicity rates when combined with immunotherapy.

Biopsy specimens pre- and post-treatment of either arm IDA or arm COBI were examined for expression of the cyclin dependent kinase inhibitor p21, a marker of p53 activity, tumor-specific HLA-A/B/C, and immune signature data. In arm IDA, there was a trend, although not statistically significant, towards increased p21 expression. Active p53 induces p21 expression, resulting in cell cycle arrest^27^, which supports our hypothesis that adding idasanutlin to these patients’ anti-cancer treatment would result in senescence. However, the immune signature data, while highly exploratory due to our limited sample size, did not show an increase in chemokine gene scores in the IDA treatment arm. Although this could be related to the small sample size, it is also possible that high p21 levels in this case are not functioning to promote the SASP, especially since we did not see changes in T cell or chemokine scores. Finally, patients with partial response or stable disease in arm IDA showed higher baseline tumor-specific HLA-ABC levels and trends towards increases in expression post-treatment.

In arm COBI, immune signature data showed significant post-treatment decreases in M2 macrophage and monocyte abundance. M2 macrophages are immunosuppressive and have been associated with progressive disease in several cancer types^28^. Moreover, MEK activation has been shown to mediate recruitment of immunosuppressive myeloid cells through expression of CXCL1 and CXCL2 in preclinical TNBC studies, a process that is substantially reduced with MEK inhibition^29^. Cobimetinib, as anticipated, resulted in a decrease in Ras/MEK gene scores, although those results were not statistically significant.

One patient showed an exceptional response that persisted for over 3 years upon follow-up. The patient underwent treatment with idasanutlin and atezolizumab for 14 months but discontinued due to patient-initiated withdrawal from the trial, citing symptom burden, including fatigue and neuropathy. Of note, this patient demonstrated conserved baseline expression of tumor-specific HLA-ABC ( > 75% of tumor cells), an apparent rarity in this patient population with a slight increase after idasanutlin lead-in. Tumor-specific HLA-ABC has been shown to be required for response to immune checkpoint inhibitors^30–32^, representing a potential biomarker of response to ICI therapy in this patient population. We did not observe an increase of p21 after idasanutlin lead-in in this patient, which could suggest that the patient was primarily responding to atezolizumab.

In early studies of metastatic TNBC, there were ongoing trends towards response to treatment in patients with higher CPS (for pembrolizumab) and TPS (for atezolizumab) scores^33–35^. However, only pembrolizumab was eventually approved in combination with chemotherapy for first-line treatment of metastatic TNBC for patients with CPS scores >/=10, while atezolizumab failed to improve PFS or OS when combined with paclitaxel^5,36^. Although ICIs have yet to be approved in the ER positive patient population, their success in TNBC makes it worth exploring alternative combinations with molecularly targeted therapies. In this study, there was one patient in arm IDA with an exceptional duration of response to treatment, but it is unclear if she responded primarily to atezolizumab or to combination therapy. We have yet to identify a biomarker that predicts patients who will be exceptional responders to immunotherapy, although the CPS score is often used for pembrolizumab in various cancers. Several retrospective analyses show that HLA class I and II expression as well as heterozygosity across HLA-ABC loci are associated with patients with improved overall survival when treated with ICIs; however, these studies did not include breast cancer patients^37,38^. The patient with an exceptional treatment response did have conserved baseline HLA-ABC expression in this study, representing an intriguing area of further exploration.

Larger phase II and III studies will be needed to explore combination therapeutic options with ICIs and MEK inhibitors or MDM2 antagonists in the future in ER+ tumors with the goal of enhancing the response to immunotherapy in these traditionally immunotherapy-insensitive tumors. Based on the results from our limited patient cohort, this is certainly a combination treatment strategy that warrants further investigation.

## Methods

### Patients

Adult patients eligible for enrollment had clinical stage IV invasive mammary carcinoma (IMC) or unresectable locoregional occurrence of IMC that was ER/PR-positive ( > 1% of cells) by IHC and HER2 negative (by IHC or FISH), previous exposure to an aromatase inhibitor (AI) or a selective estrogen-receptor modulator/downregulator (SERM or SERD) plus a CDK4/6 inhibitor, and measurable disease as defined by Response Evaluation Criteria in Solid Tumors (RECIST) version 1.1 criteria, ECOG performance status of 0 or 1, and adequate organ function. Female patients of childbearing potential were required to use at least two methods of acceptable contraception from 15 days prior to their first study treatment until at least 5 months after their final dose of study drugs.

Key exclusion criteria were: prior therapy with anti-PD-L1/anti-PD1 antibodies, MEK inhibitors, or MDM2 antagonists, more than 3 prior lines of chemotherapy in the metastatic setting, presence of a medical condition that would make the patient ineligible for ICI therapy (ex. receipt of any organ transplantation or autoimmune disease), use of corticosteroids or immunosuppressive medications to treat an autoimmune condition, pregnancy or breastfeeding, or known risk factors for ocular toxicity. Patients should also not have known hypersensitivity reactions (NCI-CTCAE grade 3 or greater) to monoclonal antibodies or a history of anaphylaxis. Use of medications, foods, or supplements known to be strong or moderate CYP3A4 enzyme inducers or inhibitors had to be stopped at least 7 days prior to cycle 1 day 1 of study treatment, and patients were excluded if they required anticoagulation therapy with an oral vitamin K antagonist.

Patients with brain metastases were permitted to enroll in the study if their brain metastases had been treated locally and were clinically stable for 2 weeks prior to study enrollment without evidence of interim CNS disease progression, were asymptomatic, were not in the midbrain, pons, or medulla, and had no history of intracranial or spinal cord hemorrhage. Patients also needed to be either off steroids or on a stable decreasing dose of 10 mg prednisone equivalent or less at the time of study enrollment.

### Trial design

In this open-label, two-arm phase Ib/II study, patients were assigned to receive atezolizumab in combination with either the MEK inhibitor cobimetinib (arm COBI) or the MDM2 antagonist idasanutlin (arm IDA). Patients were assigned to arm COBI if they had *TP53*-mutated disease and were assigned to arm IDA if they had *TP53-*wild type (wt) disease. *TP53* mutation status was determined either from previous tissue NGS or cfDNA testing results or from NGS testing of archival tissue that was provided to the study sponsor at the time of patient enrollment. One patient did have NGS results that were amended to report *TP53*-wt status, and those results were confirmed using a Guardant360 liquid biopsy prior to starting treatment. Patients in each arm underwent a 15-day lead-in treatment period with either 60 mg cobimetinib or their assigned dose of idasanutlin, (planned dose ranges of 50–200 mg) after which they underwent a tumor biopsy and blood collection prior to the initiation of atezolizumab. Drugs were then administered as follows: atezolizumab 840 mg on days 1 and 15 every 28 days in both arms; arm COBI: cobimetinib 60 mg PO daily on days 1–21 every 28 days, and arm IDA: idasanutlin on days 1–5 every 28 days (study schema provided in Supplemental Fig. 1).

The phase Ib dose-escalation schedule of idasanutlin used a standard 3 + 3 dose escalation scheme with doses as follows: 100 mg, and 150 mg, 200 mg. 100 mg was dose level 1, and dose level -1 was 50 mg. Dose limiting toxicities were assessed during the first six weeks of treatment and were defined by study protocol. Maximum tolerated dose (MTD) of idasanutlin was defined as the highest dose tested in which a dose-limiting toxicity was experienced by 0 out of 3 or 1 out of 6 patients among the dose levels. The phase II portion of the study planned to use the idasanutlin MTD to assess tolerability and efficacy. Treatment continued until one of the following occurred: confirmed disease progression, unacceptable toxicity, patient withdrawal of consent, or the decision of the treating physician to stop treatment.

### Assessments

Patients were monitored with staging CT scans and nuclear medicine bone scans every 8 weeks while on the study. Adverse events were monitored throughout the study using the NCI CTCAE v5.0, with the total number of grade 3 or higher adverse events reported here. Patients also had history assessment, physical examination, and routine blood work obtained at minimum on day 1 of each cycle. Ophthalmologic examinations were also performed prior to treatment, on day 1 of cycle 2, and then every 12 weeks until the end of treatment.

For correlative studies, cfDNA samples were collected in Streck tubes on C1D1, C1D15, C3D1, and at the end of treatment. Tumor biopsies were collected prior to initiating treatment with cobimetinib or idasanutlin and again after 15 days on treatment with those agents prior to the initiation of atezolizumab, one core was formalin-fixed and paraffin embedded, and the remaining cores were snap-frozen. The second biopsy was always requested to be taken from the same site as the initial biopsy sample, although the second biopsy site could be taken from the metastatic lesion closest to the original biopsy site at the discretion of the proceduralist if the initial biopsy site was not amenable to a second biopsy (this one done for one patient in this study).

### Immunohistochemistry analysis

Paired formalin-fixed paraffin embedded tissue with viable tumor content on was available for 8 patients, 4 in each arm. Chromogenic and fluorescence IHC procedures were performed on 4 μm sections. After deparaffination, antigen retrieval was performed with citrate buffer pH 6. Endogen peroxidase were blocked and protein block was applied. Sections were then incubated with the primary antibody overnight at 4 °C. For p21 (12D1 cat#2947 Cell Signaling at 1:400), visualization utilized Envision (Agilent Technologies, Santa Clara, CA), with DAB as the chromogen (Agilent Technologies) and hematoxylin as the counterstain. For CK (AE1/AE3 cat#CM011ABC Biocare at 1:400) HLA-A/B/C (C6 cat#sc365495 Santa Cruz at 1:650) multiplex fluorescence IHC, sections were then incubated with the secondary antibody and TSA reagent applied according to manufacturer’s recommendations. The procedure was repeated for the second primary antibodies and then counterstained with DAPI for nuclei identification.

Whole slide images were digitally acquired using an AxioScan Z1 slide scanner (Carl Zeiss) at 20×. Automated quantification was performed via a pathologist-supervised machine learning algorithm using QuPath software^39^. Cell segmentation was determined with the built-in cell detection algorithm on DAPI. Tumor cells were defined by training an object classifier on annotated regions from control tissue and tumor samples using panCK expression and subcellular characteristics. For p21 and HLA-A/B/C we combined the high and low expressing areas to create a bimodal distribution histogram of intensity measurements to set the threshold to define a positive cell. Once the algorithm was performing at a satisfactory level, it was used for batch analysis. For cases with low, heterogeneous, or null CK expression in which the classifier’s performance was not optimal, tumor areas were manually annotated. Out-of-focus areas, tissue folds, necrosis, normal breast, and in situ carcinoma were excluded from the analysis. A pathologist visually assessed each sample for the correct performance of the algorithm.

Determination of stromal tumor-infiltrating lymphocytes (TILs) was performed according to the International TILs Working Group Guideline using hematoxylin and eosin (H&E) whole slide images^40^.

### RNA sequencing and analysis

Tumor tissues were dissected from frozen tissue sections and RNA extraction performed using the Maxwell-16 LEV simply RNA tissue purification kit (Promega). RNA quality was assessed using the 2200 TapeStation (Agilent). DNase-treated total RNA having at least 30% of the RNA fragments with a size >200 nucleotides was used to generate RNA access libraries (Illumina) following manufacture’s protocols, Library quality was assessed using the 2100 Bioanalyzer (Agilent) and quantified using KAKA Library Quantification Kits (KAPA Biosystems). Pooled libraries were sequenced at Paired-End 150 bp on the Illumina NovaSeq 6000 targeting an average of 50 M reads per sample. Sequencing reads were mapped to human reference genome GRCh38 and quantified using Salmon 1.10.1 mapping-based mode. QC for read alignment and mapping was evaluated with MultiQC for alignment rate and fragment length distribution. Transcript-per-million values were extracted from Salmon output and used to assess the global quality and replicability of the RNA-seq data set and exported for downstream analyses.

CIBERSORT analysis was performed on RNA-seq transcript-per-million level data generated above (https://cibersort.stanford.edu/). CIBERSORTx was used in relative mode with 500 permutation and the LM22 reference matrix.

### Gene signature score calculation

The Ras-ERK activation signature was comprised of 57 upregulated genes, as reported previously^41^, and the resulting signal intensities were summed to generate the Ras-MEK pathway scores. For the chemokine gene score, the TMPs of CCL5, CXCL9, CXCL10, and CXCL1, which are T cell homing cytokines^19^, were log-2 transformed and were summed for each pathway to generate the chemokine score.

### ctDNA sequencing and analysis

Tumor tissues were dissected from frozen tissue sections and DNA extraction was performed using the Maxwell-16 LEV DNA purification kit (Promega). DNA quality was assessed using the 2200 TapeStation (Agilent). Tumor-informed ctDNA analysis was performed using Haystack MRD (Haystack Oncology, Quest Diagnostics, Secaucus, NJ). Following whole exome sequencing of tumor tissue and matched normal specimens (Tempus AI Inc.), up to 50 patient-specific somatic mutations were selected and incorporated into the design of personalized multiplex PCR-NGS assays to assess ctDNA levels in plasma. Cell-free DNA was purified from plasma samples using the KingFisher Apex (Thermo Fisher Scientific) with cfPure MAX extraction reagents (BioChain Institute) and quantified using the Cell-Free DNA ScreenTape on the Agilent 4200 TapeStation (Agilent). Up to 50,000 genomic equivalents were used for library preparation, patient-specific target amplification, and paired-end sequencing on a NovaSeq 6000 sequencing instrument (Illumina) with Haystack MRD, a modified version of the Safe-Sequencing System error-reduction technology for the detection of low frequency mutations^42,43^. Sequencing data was demultiplexed using Illumina bcl2fastq in BaseSpace and analyzed with Haystack Oncology’s MARS (MRD Analysis and Results Software) suite of pipelines to report the presence and associated levels of ctDNA in plasma samples.

### Study oversight

The study was developed by the Vanderbilt-Ingram Cancer Center (VICC) Breast Cancer Research Team. The VICC Data and Safety Monitoring Committee (DSMC) provided oversight for patient safety and data monitoring throughout the course of the study. This study was conducted in accordance with the Good Clinical Practice guidelines and the Declaration of Helsinki, and the study protocol was approved by the Vanderbilt University Institutional Review Board. All patients provided written informed consent before enrollment, in agreement with the approved protocol. ClinicalTrials.gov registration number: NCT03566485.

### Statistical analysis

The primary endpoint for the phase II portion of this study was progression-free survival (PFS). Simon’s Minimax design was used to estimate sample size, with stage I and II sample sizes of 16 and 17, respectively (maximum total of 33 patients per arm) to ensure that the total number of patients accrued to each arm was minimized. Assuming an exponential survival model based on historical controls, “response” for this study was estimated as PFS at 16 weeks (yes/no). The futility boundary for stage I was set at 4 responses, so accrual would have been stopped after data from the 16^th^ patient were available if the number of responders were below the threshold. If 13 or more responses were observed at the end of the trial, then clinical benefit of cobimetinib and atezolizumab or idasanutlin and atezolizumab would have been concluded. This design had a 4.5% type I error rate at a response rate of 25% and 90% power if the true “response” rate is 50% or higher.

Secondary endpoints included overall response rate (ORR), which was defined as the percentage of patients achieving complete response (CR) or partial response (PR) by RECIST v1.1 criteria and clinical benefit rate, defined as lack of disease progression at 6 months. Safety was also evaluated with a plan to present all adverse events with descriptive statistics.

For correlative studies, biomarkers were characterized as continuous or dichotomous variables. A paired t-test was used to determine if the proposed endpoints were significantly increased in paired biopsy in either arm.

## Supplementary information

Supplementary Figure 1

## Data Availability

The data generated in this study are available upon request from the corresponding author.
