# Supplementary figures and images for: A phase Ib/II trial of atezolizumab with cobimetinib or idasanutlin in metastatic estrogen receptor positive breast cancer
Source: NPJ Breast Cancer. 2025 Jun 21;11:61. doi: 10.1038/s41523-025-00773-4 (PMC12182567; doi:10.1038/s41523-025-00773-4)

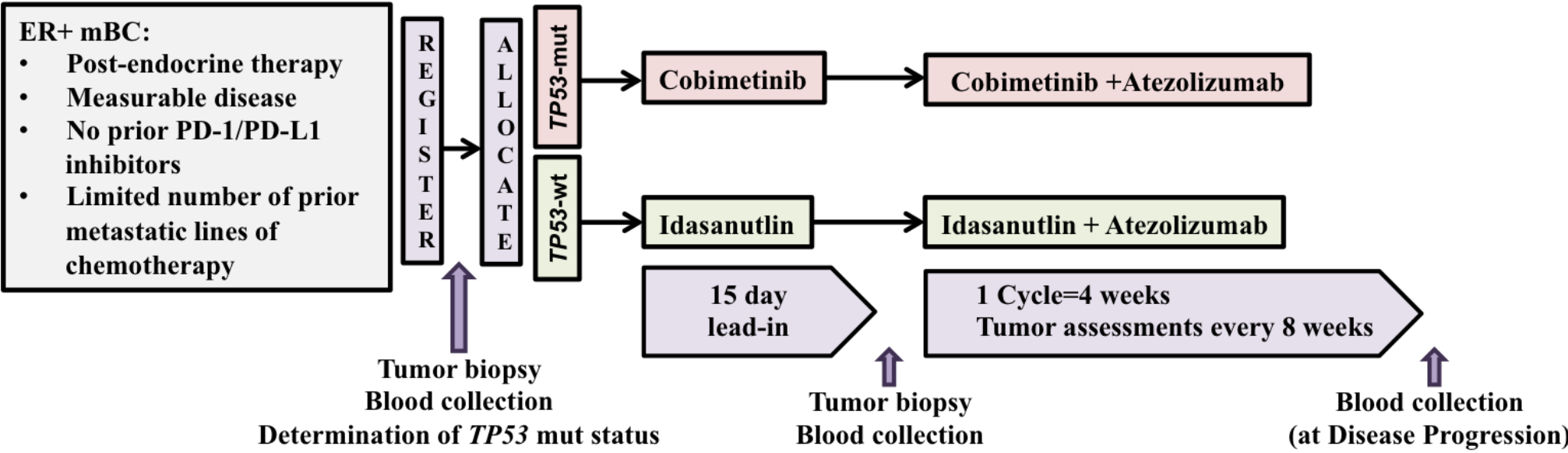

**Supplemental Figure 1:** Study schema. mut: mutant. wt: wild-type.

Supplement: Supplementary file 1 — Supplementary Figure 1 [file 41523_2025_773_MOESM1_ESM.pdf]
